# Effects on 24-h physical behaviors and heart rate variability during sleep of a co-created workplace intervention to promote recovery in office workers with flexible work

**DOI:** 10.1186/s44167-026-00107-0

**Published:** 2026-06-28

**Authors:** Johanna Edvinsson, Svend Erik Mathiassen, David M. Hallman

**Affiliations:** Department of Occupational Health, Psychology and Sports Sciences, Faculty of Health and Occupational Studies, University of Gävle, Gävle, Sweden

**Keywords:** Recovery from work, Physical activity, Sleep, Health promotion, Workplace intervention, Compositional data analysis

## Abstract

**Background:**

As flexible work arrangements (FWAs) become more common among office workers, the challenge of maintaining a healthy balance between work and recovery increases. However, studies addressing workplace interventions to promote recovery in FWAs are sparse. This study examined the effects of a co-created workplace intervention on the 24-hour composition of physical behaviors and recovery during sleep among office workers with FWAs.

**Methods:**

A controlled intervention study was performed in a large governmental organization offering FWAs. Office workers from one unit (*n* = 27) participated in, (1) an individual-level course on work strategies and (2) a workgroup-level workshop to develop common rules and routines for FWAs. These activities were expected to reduce work demands, facilitate detachment after work, and promote healthier 24-hour physical behavior patterns and improve recovery during sleep. Employees from a comparable unit were included as a control group working as usual (*n* = 21). Physical behaviors at baseline and at a 12-month follow-up were assessed in both groups using 24-hour accelerometry for three days, together with heart rate variability indicators of recovery during sleep. We calculated time used in physical activity, inactivity and sleep in a Compositional data analysis framework, and analyzed intervention effects on these behaviors and heart rate variability indicators using repeated-measures MANOVA.

**Results:**

The intervention led to, on average, 36 min more sleep per night, compared to 23 min less sleep in the control group, and the effect size was large (F = 10.87, *p* < 0.01, ηₚ² = 0.28). The intervention had limited effects on physical activity relative to inactivity, and on heart rate variability during sleep (interaction between time and group: *p* > 0.05).

**Conclusions:**

An intervention combining intervention activities at the individual and workgroup levels led to longer sleep time, indicating a behavioral effect that may promote recovery and health. The intervention did not, however, affect physical activity behaviors while awake, or heart rate variability indicators of recovery during sleep. These findings suggest that interventions targeting individual and collective work practices may influence physical behaviors and recovery. Further studies are needed to examine long-term effects in other groups of workers with flexible work.

## Background

The increasing prevalence of flexible work arrangements (FWAs) and use of information and communication technology (ICT) provide both opportunities and challenges for creating and maintaining a healthy balance between work and recovery, especially among office workers [1]. Office workers commonly perform desk-based work using ICT, such as computers, and this work is characterized by varying degrees of flexibility regarding when, where, and how it can be performed [2]. Thus, FWAs can be an opportunity for employees to tailor their work time to fit their personal needs and lifestyles, such as engaging in recovery-promoting behaviors [3, 4].

Recovery refers to the “unwinding and restoration processes during which a person’s strain level that has increased as a reaction to a stressor or any other demand returns to its prestressed level” [5] (p. 366). In mentally demanding jobs, such as office work, psychological detachment from work is important for recovery, since it allows employees to mentally disengage from work-related demands during non-work time [6]. Psychological detachment refers to “the subjective experience of leaving work behind, to ‘switch off’ and forget about work during nonwork time” [5] (p. 4). Meta-analyses, mainly based on cross-sectional studies and self-reported data, suggest that detachment has a potential to facilitate both psychological, physiological, and behavioral recovery indicators [7, 8]. For example, detachment from work is associated with less exhaustion, better well-being, lower levels of self-reported need for recovery, and better sleep [7, 8] and it may allow engagement in health-promoting physical activity [9, 10].

While showing a potential to promote recovery, FWAs also pose challenges to factors associated with recovery [11]. Office workers with FWAs may experience blurred boundaries between work and private life and challenges to psychological detachment [7, 12]. For example, FWAs can increase job demands by fostering an “always-on” culture in a workgroup, where employees are, or feel, expected to be constantly available for work, which can challenge opportunities to detach from work [13, 14]. FWAs also allows for work during nonwork time, which can lead to longer working days and less time for physical activity during the day [15, 16], sleep of shorter duration or lower quality [17], and thus a lack of recovery [18, 19] which can be detrimental to health [9, 20].

Research has increasingly emphasized the importance of considering the entire 24-hour composition of physical behaviors, including sleep, when addressing recovery and health [21, 22]. This is particularly relevant for workers in sedentary occupations, such as office work. Adults are generally recommended to replace physically inactive time (i.e., lying, sitting, standing still) with physical activity [21, 23], and to sleep 7–9 h per night [24]. Studies indicate that the physical behavior composition is associated with outcomes of relevance to recovery such as affective states [25], self-reported need for recovery [26] and heart rate variability (HRV) [27]. However, findings are still limited, and it remains unclear whether workplace interventions can influence the 24-hour composition of physical behaviors among office workers with FWAs [28], and, in that case, which components in an intervention that effectively lead to positive results.

In addition to time spent in different physical behaviors, the ability to physiologically unwind during sleep is important for recovery. HRV reflects autonomic nervous system regulation with a high HRV indicating greater parasympathetic activity and better recovery, and a low HRV indicating physiological strain or insufficient recovery [29]. In particular, HRV during sleep provides information on physiological recovery processes [30, 31]. Although HRV is an established indicator of stress and recovery, much of the evidence comes from laboratory studies, and less is known about its sensitivity to interventions in real-life workplace settings [32]. In addition, previous intervention studies have largely relied on self-reported outcomes of the intervention, rather than on measurements of physiological recovery [28].

Employers offering FWAs may facilitate engagement in recovery activities by providing employees with support in detaching from work [33]. Some studies have focused on implementing interventions at the individual level to promote detachment in FWAs [28] supported by systematic and narrative reviews suggesting that employers offering FWAs need to focus on detachment when promoting employee recovery and well-being [7, 13, 34]. Thus, employers can provide work strategies for better management of work demands and the employees can be equipped with resources such as boundary management skills, effective planning, and task prioritization [13, 34]. At the organizational level, employers can strengthen such initiatives by clarifying reasonable expectations regarding availability during nonwork hours and implementing policies to prevent continued work [7]. For instance, they can facilitate an allowing culture regarding availability expectations and norms in workgroups [13].

Implementation theories and principles for workplace interventions emphasize the importance of involving the target group when identifying needs and developing intervention activities [35]. Such participatory approaches can help ensure that interventions are relevant, feasible, and adapted to the specific work context [35, 36]. Health-promoting workplace interventions are also more likely to be effective if activities are combined at multiple organizational levels (i.e., organizational, group and individual levels) [28, 37]. However, to our knowledge, no study has examined the effects of integrated intervention activities at multiple organizational levels on recovery indicators [28], including whether interventions intended to improve recovery through better detachment influence physical behaviors and sleep among workers with FWAs [28, 38].

We aimed to address this research gap by conducting a research project examining the effects among office workers with FWAs of a co-created workplace intervention on psychological, behavioral and physiological recovery indicators. Another study from the project indicated that the intervention had positive effects on self-reported psychological recovery among employees with higher initial need for recovery [39]. The present study complements these findings by addressing the effect of the same intervention on 24-h physical behaviors and physiological recovery. The aim was to examine the extent to which the intervention influenced time used in active and inactive behaviors and sleep based on 24-hour accelerometry recordings of physical behaviors, as well as physiological indicators of recovery, measured by HRV during sleep. Specifically, the research questions (RQ) were:

- *RQ*1: To what extent does the workplace intervention change the 24-hour composition of physical behaviors in terms of sleep vs. waking time and, during waking time, physically active time vs. inactive time?
- *RQ*2: To what extent does the workplace intervention change recovery during sleep, as indicated by HRV?

We expected the intervention to increase time in sleep relative to time awake as well as time with physical activity relative to inactivity, while also promoting recovery during sleep.

## Methods

### Study design

This study is part of the co-created research project “Flexible Work: Health-promoting Interventions for Sustainable Digitalized Work,” conducted in a Swedish governmental agency. The research project comprised three phases, i.e. (1) a comprehensive questionnaire focusing on the work environment and health among all office workers with FWAs within the organization (*n* = 4900) [19, 40], (2) focus group interviews with representants from all divisions of the organization identifying suggestions for interventions (*n* = 45) [41], and (3) designing and implementing a workplace intervention in one department in the organization [39, 42] based on results in the previous steps.

**Fig. 1:**
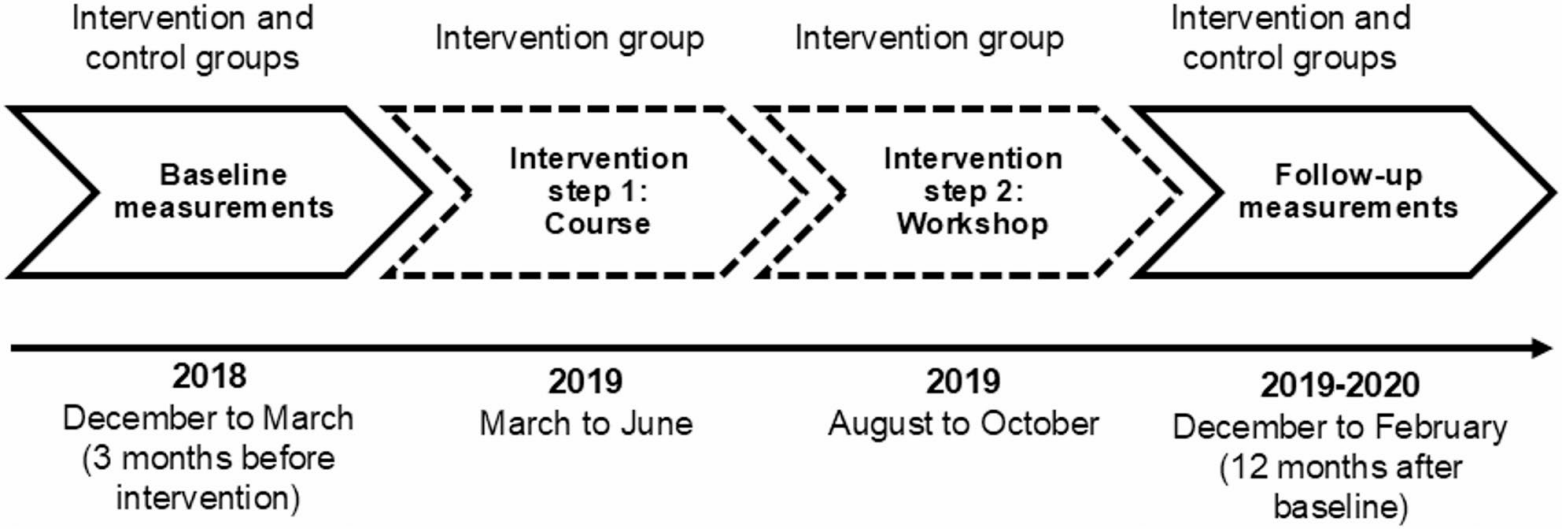
Timeline of intervention activities and data collection

The present study is based on the third phase and used a quasi-experimental design with two non-randomized groups in the agency: an intervention group and a control group, working in two departments within the same division, but situated at different geographical locations. The intervention was implemented in two steps: (1) an individual-level course and (2) a workgroup-level workshop (described in more detail below). All participants in the intervention group received both intervention activities. The control group was aware of the intervention activities but received no intervention. Data were collected at baseline (3 months before the intervention) and at 12 months after baseline (Fig. 1). Ethical approval was obtained from the Swedish Ethical Review Authority (2017/528), and all participants provided written informed consent before entering the study.

### Intervention

The intervention was developed through a co-creation process involving employees, employer representatives, and researchers. The intervention activities were informed by needs identified by employees and the management and were selected based on their presumed relevance, feasibility, and potential effectiveness in the work context [41]. The intervention focused on strengthening individual and collective work practices in flexible work, such as planning and prioritizing work, managing interruptions and ICT use, clarifying availability expectations, improving meeting routines, and developing shared rules and routines within the workgroup. Based on theory and previous research on recovery, these activities were expected to reduce work-related demands and facilitate psychological detachment from work, which would result in more time for physical activity, longer duration of sleep and improved physiological and self-reported indicators of recovery [7, 13, 28, 34, 43].

The intervention consisted of two steps: (1) An individual-level course intended to enhance the worker’s ability to use digital tools to better plan and prioritize work, minimize work interruptions, and create structure and control over the work. (2) A workgroup-level workshop focused on developing common rules and routines for flexible work, such as clarifying expectations for availability.

The individual-level course was delivered to 14 groups of eight to 14 participants each. Participants were offered several opportunities for when to have the course and signed up for the option that best suited their availability. Group sizes were based on practical feasibility. The course included three sessions: 1) a four-hour webinar introducing digital tools; 2) an eight-hour on-site seminar on how to use these tools to reduce interruptions, organize and prioritize tasks, and finish the day with a sense of control; and 3) a four-hour follow-up webinar to reinforce the strategies. The course took place from March to June 2019 and was led by an external educator from a consultant specialized in personal work efficiency. Before each session, participants received preparatory information and could contact the educator with questions. The educator was also available after each session.

The workshop was delivered to eight workgroups (corresponding to units within the department) of 15–27 participants each as well as their managers between August and October 2019. The workshop for one additional work group was cancelled due to a booking error. The workshop consisted of a single six-hour, in-person session aimed at strengthening the workgroup culture by developing common rules and routines in flexible work. The session was led by a moderator familiar with the organization and used a structured method [44] adapted and pilot-tested within the organization before the intervention. The workshops resulted in action plans specifying each rule or routine, its purpose, how it would be applied, and who was responsible for finalizing it and when this should have happened. Examples of rules developed and agreed upon during the workshops were routines for how to signal availability to colleagues, and what to expect regarding whether and when employees could respond to work-related communication outside regular working hours.

The implementation process has been reported elsewhere [42]. Approximately 94% of the eligible employees participated in the course and 82% in the workshop. The course was generally perceived as being both satisfactory and relevant to work, and most participants also reported high engagement. Similar experiences were reported for the workshop; about one-third of the participants reported that they kept working with the developed action plan [42].

### Participants

Representatives from the organization’s Human Resources department were responsible for recruiting participants. The intervention and control groups were part of the same organizational division, which included 12 departments in total. Two of these departments were selected, one for the intervention group (which had expressed interest in participating) and one for the control group working as usual (they were aware of the intervention but did not receive it). The intervention group comprised 183 employees in total, while the control group comprised 161 employees. Of these eligible participants, 48 office workers with FWAs expressed their interest in participating in the technical measurements, met the inclusion criteria and were available for the baseline measurements (intervention group *n* = 27, control group *n* = 21). Of those, 42 participants also participated in the follow-up measurement (intervention group *n* = 26, control group *n* = 16). Inclusion criteria were that participants needed to have FWAs in the form of flextime (i.e., flexibility regarding work time within fixed core hours) or trust-based working hours (i.e., full autonomy over when they worked), that they participated in the intervention activities and that technically measured physical activity and heart rate data were available.

### Data collection

Baseline measurements were performed three months before the intervention, and follow-up measurements were performed 12 months after baseline, after completion of both intervention activities (Fig. 1). Data were collected for three consecutive days during one week of work, which has been shown to be sufficient to determine most physical behavior variables with a fair reliability during typical work weeks in office workers [45–47]. The data collection started with individual sessions, during which a research team member provided details about the study and the data collection, informed about the measurements and attached the accelerometer and the devices measuring HRV. Following data collection, participants detached the measurement instruments themselves, and a member of the research team collected the instruments and downloaded the recorded data.

### Measurements

#### Physical behaviors

24-hour physical behaviors, including time spent in physical activity and inactivity, and sleep, were measured using an accelerometer (ActiGraph GT3X+, Florida, USA) combined with a diary reporting self-reported sleep and waking times. We used the Axivity software (Axivity AX3, Axivity Ltd., Newcastle UK) to initialize the accelerometer before the measurement and to download the data afterwards. During the start-up session, the accelerometer was attached to the skin on the front of the right thigh using double-sided adhesive tape and waterproof adhesive film. As a reference, we asked participants to stand upright followed by a jump, and this procedure was performed at the start and at the end of the measurement period [48]. In the diary, participants were asked to fill in the times they fell asleep and woke up (used to identify the sleep periods), the times they detached the accelerometer before contact with water (e.g., shower or bath) and re-attached it afterward (indicating non-wear time), and the time they detached the accelerometer after data collection.

#### Physiological recovery

Beat to beat heart rate (HR) was measured using a heart rate monitor (Firstbeat Technologies Ltd., Jyväskylä, Finland) and used to assess HRV as an indicator of physiological recovery. The monitor was attached on the right side of the upper chest under the collarbone, and one on the left side, on the lower chest, on the rib cage.

#### Questionnaire

In parallel with the technical measurements, the participants were asked to answer a questionnaire including demographic characteristics and items related to recovery, work-life balance, and health (parts of the results are published elsewhere [39, 42]. Baseline data were collected on sex (female, male), marital status (single, living apart, married/partnership), children at home (no children, one or more children at home), organizational position (manager/team leader, employee) employment rate (100%, less than 100%), agreed and actual working hours (hours), height (centimeters), weight (kilograms), and psychological detachment. Psychological detachment was measured with the Swedish version of the Recovery Experience Questionnaire [49, 50], including four items (e.g., ‘After work, I forget about work’). Items were rated on a 0–4 scale and averaged (Cronbach’s α = 0.86), with higher scores indicating greater detachment. The intervention effects on detachment and need for recovery have been published elsewhere [39].

### Data processing

#### Physical behaviors

The accelerometer data were processed using Acti4 (Acti4, The National Research Center for the Working Environment, Copenhagen, Denmark, and BAuA, Berlin, Germany) to identify different types of physical behaviors [51]. After subtracting time in sleep, as determined from the diary, we categorized time awake into active (i.e., walking, stair-walking, running and cycling) and inactive (i.e. sitting/lying and standing) time. The time series of classified behaviors were imported to Spike software version 9 Windows (Cambridge Electronic Ltd., Cambridge, UK) along with the diary data, and HRV (described below) for visual inspection and additional processing, including averaging data across all days of measurement for each participant.

We used compositional data analysis (CoDA) for analyzing proportions of time spent on active and inactive physical behaviors and sleep. CoDA accounts for daily time-use data being constrained to a total of 24 h and considers the interdependence between behaviors during this time. According to standard CoDA procedures, we created two isometric log-transformed ratios (ILRs) [52, 53]: one measuring time in sleep relative to time awake (ILR1), and one, within time awake, measuring time being physically active relative to time being inactive (ILR2). In case of missing daytime data, we assumed that the proportions of behaviors during the unknown time were identical to the proportions during the observed time awake.

#### Physiological recovery

The HRV recordings were obtained from Firstbeat Bodyguard2 (Firstbeat Technologies Ltd., Jyväskylä, Finland). The data was exported to Firstbeat SPORTS 4.7 software (Firstbeat Technologies Ltd., Jyväskylä, Finland), where an artifact correction script was applied to filter out incorrectly recorded heartbeats. Two researchers visually screened data for errors and excluded periods with excessive artifacts and missing data. We then calculated HRV variables that can indicate overall recovery, by using the root mean square of successive differences between normal-to-normal intervals (RMSSD), which reflects parasympathetic activity [29], and the standard deviation of the inter-beat intervals of normal sinus beats (SDNN), which reflects both sympathetic and parasympathetic activity [29, 54]. Sleep represents a period with minimal external stressors. Thus, measuring HRV during nocturnal sleep provides a valid indicator of autonomic nervous system modulation and adaptability, particularly reflecting parasympathetic activity [29]. The RMSSD and SDNN were calculated based on the means across all three days of measurements.

### Statistical analysis

Statistical analyses were performed using the Statistical Package for the Social Sciences (SPSS, version 27.0, IBM, Armonk, NY, USA). Baseline demographics of the study sample and the physical behavior and recovery variables at baseline and follow-up are presented with means and standard deviations (SD) for continuous variables and frequencies and percentages for categorical variables.

To examine the intervention effects on the 24-hour composition of physical behaviors (RQ1) we used repeated-measures multivariate analyses of variance (RM-MANOVA). The model included ILR1 (time in sleep relative to time awake) and ILR2 (time being physically active relative to being inactive). Another model, examining RQ2, included HRV indices (SDNN and RMSSD) as indicators of recovery during sleep as dependent variables. Then, we proceeded with repeated measurements univariate analyses of variance (RM-ANOVA) for each dependent variable. These univariate models included main effects of time (baseline and follow-up) as a within-subjects factor, and group (intervention and control) as a between-subjects factor, as well as the interaction (group x time). We determined effect sizes using partial eta square (ηₚ²) and classified them according to Cohen’s reference values for small (~ 0.2), medium (~ 0.5), and large (~ 0.8) effects [55]. The statistical tests were run first without and then with covariates, i.e. baseline data for body mass index (BMI), age, sex, and children at home, since these variables have been associated with physical activity and/or HRV during sleep [56–58]. BMI was calculated by dividing the participant’s weight in kilograms by their height in meters squared.

## Results

### Descriptive statistics of the study sample

The descriptive statistics of the study sample at baseline (Table 1) showed that the participants in the intervention group were, on average, slightly older than those in the control group (49 vs. 46 years). There were fewer women in the intervention group (19% vs. 43%). More participants in the intervention group had children living at home (67% vs. 57%). A smaller proportion of participants in the intervention group had a leadership position (15% vs. 38%). Almost all participants worked full-time, defined as 40 h per week, while two participants worked part-time (75% of full-time employment). Both groups had an agreed weekly working time of 40 h, but actual working hours were slightly higher in the intervention group (42 h vs. 41 h). The groups had similar levels of detachment at baseline (around 2.5 on the 0–4 scale).

**Table 1:** Descriptive statistics of the intervention and control groups at baseline

	Intervention group (*n* = 27)		Control group (*n* = 20*)	
	Mean (SD)	*n* (%)	Mean (SD)	*n* (%)
Age (years)	49.4 (10.8)		46.2 (10.8)	
*Sex*				
Men		22 (81)		11 (55) 9 (45)
Women		5 (19)		
*Marital status*				
Single		4 (15)		1 (5)
Living apart		2 (7)		0 (0)
Married/partner		21 (78)		19 (95)
*Children*				
No children		9 (33)		8 (40)
Children at home		18 (67)		12 (60)
*Organizational position*				
Manager/team leader		4 (15)		8 (40)
Employee		23 (85)		12 (60)
*Employment rate*				
100%		25 (93)		19 (95)
Other		2 (7)		1 (5)
Agreed weekly working hours	39.7 (1.5)		40.0 (0.0)	
Actual weekly working hours	41.9 (5.8)		41.4 (3.4)	
BMI	24.4 (3.7)		26.8 (5.2)	
Psychological detachment	2.6 (1.0)		2.3 (1.0)	

BMI = body mass index. *One participant did not answer the baseline questionnaire

### Descriptive data of physical behaviors and recovery during sleep at baseline and follow-up

**Fig. 2:**
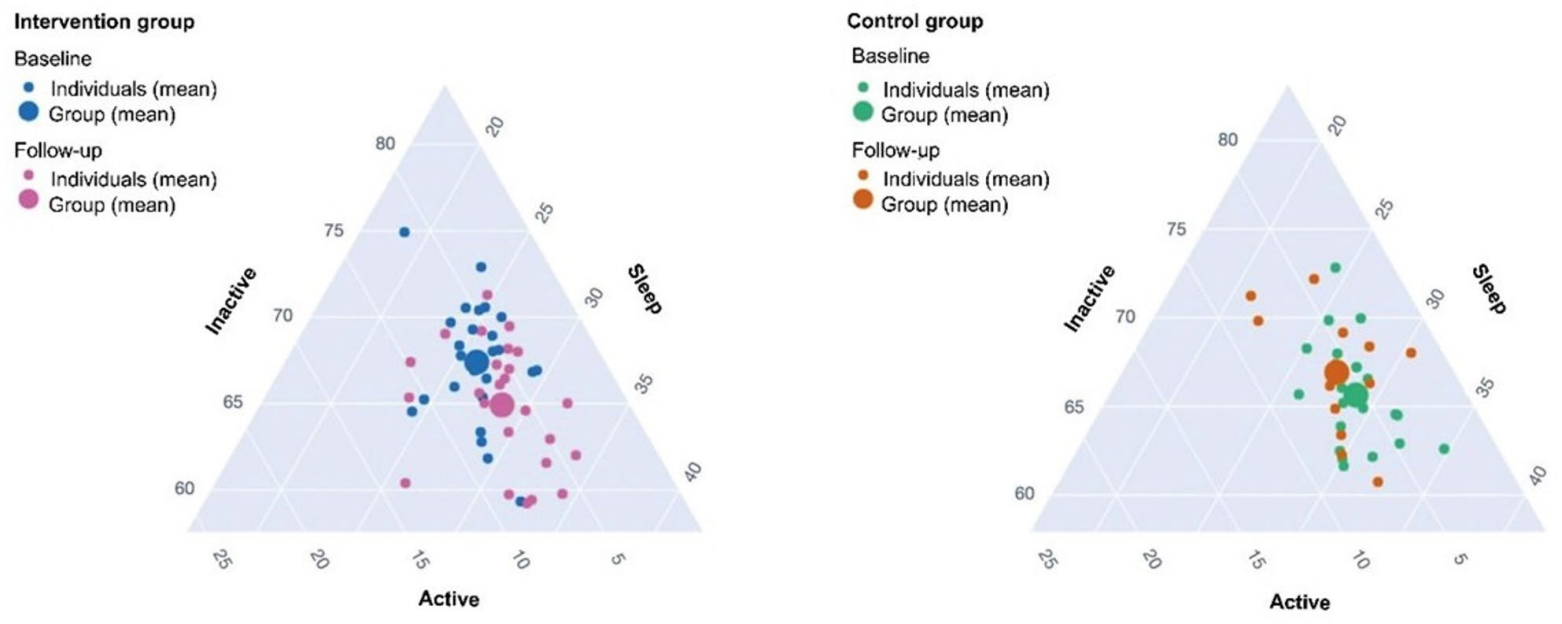
Ternary plots of 24-hour physical behavior compositions at baseline and follow-up in the intervention (left) and control groups (right). The plots illustrate 24-hour compositions of active, inactive and sleep behaviors for individual employees, as well as the averages of the groups, all expressed in percentage of time (%) at baseline and follow-up

24-hour physical behaviors and HRV variables before and after the intervention are presented in Table 2, and individual 24-hour compositions of sleep, inactive, and active behaviors are illustrated in Fig. 2. In the intervention group, sleep increased by 36 min per day (from 376 min at baseline to 412 min at follow-up), i.e. from 26.1% of 24 h to 28.6% (i.e. +2.5%). ILR1 increased accordingly. The increase in sleep was accompanied by decreases in both inactive and active time awake. ILR2 decreased slightly, indicating a small shift towards less active time relative to inactive time during waking hours. In the control group, sleep decreased by 23 min per day (from 416 to 393 min), i.e. from 28.9% to 27.3% (i.e. − 1.6%), and ILR1 decreased. Both inactive and active time awake increased, while ILR2 changed very little.

**Table 2:** Descriptive statistics (mean across days, SD) of physical behaviors, ILRs, and HRV variables (RMSSD and SDNN) before and after the intervention for the intervention and control groups

	Intervention group		Control group	
	Baseline (*n* = 26)	Follow-up (*n* = 26)	Baseline (*n* = 21)	Follow-up (*n* = 16)
	Mean (SD)	Mean (SD)	Mean (SD)	Mean (SD)
Time in sleep (min)	376 (39)	412 (48)	416 (37)	393 (48)
Time being physically active while awake (min)	94 (29)	92 (35)	79 (24)	97 (29)
Time being physically inactive while awake (min)	970 (50)	935 (50)	944 (43)	963 (49)
Time in sleep (%*)	26.1 (2.7)	28.6 (3.3)	28.9 (2.6)	27.3 (3.3)
Time being physically active while awake (%*)	6.5 (2.0)	6.4 (2.4)	5.5 (1.7)	5.8 (2.0)
Time being physically inactive while awake (%*)	67.4 (3.5)	64.9 (3.5)	65.6 (3.0)	66.9 (3.4)
ILR1**	0.20 (0.15)	0.29 (0.19)	0.36 (0.16)	0.30 (0.23)
ILR2**	− 1.69 (0.25)	− 1.67 (0.26)	− 1.79 (0.26)	− 1.80 (0.33)
RMSSD (ms)	38.2 (21.5)	34.6 (18.2)	40.9 (22.6)	39.2 (18.3)
SDNN (ms)	99.9 (28.4)	99.3 (27.7)	93.8.1 (21.1)	89.1 (21.1)

*: Percent of 24 h (1440 min)

**: ILR1 = time sleeping relative to time awake, ILR2 = time being active relative to time being inactive while awake

### Statistical analyses of the intervention effects

Results from the multivariate and univariate analyses are presented in Table 3. The RM-MANOVA of physical behaviors showed a statistically significant interaction between time and group, indicating that changes over time differed between groups in both the unadjusted (ηₚ² = 0.32, *F* = 8.11, *p* < 0.01) and the adjusted model, the latter showing a slightly stronger effect (ηₚ²= 0.40, *F* = 8.91, *p* < 0.01). The results from the RM-ANOVA showed a statistically significant change in sleep relative to time awake (ILR1) with a large effect size. The interaction between time and group had a large effect size in the unadjusted model (ηₚ² = 0.22, *F* = 10.00, *p* < 0.01), which became stronger after adjustments (ηₚ² = 0.28, *F* = 10.87, *p* < 0.01). There were no statistically significant changes in physical activity relative to inactivity (ILR2) nor on recovery during sleep (RMSSD and SDNN) (all effect sizes were small and *p* > 0.05). The results remained robust after including sex, children, age, and BMI as covariates.

**Table 3:** Intervention effects from the multivariate repeated-measures MANOVA and the univariate repeated-measures ANOVAs of physical behaviors expressed as isometric log ratios (ILR), as well as recovery (heart rate variability) during sleep expressed as RMSSD and SDNN

	Unadjusted model			Adjusted model		
	F-value	*P*-value	ηₚ²	F-value	*P*-value	ηₚ²
**Repeated-measures MANOVA**						
*Physical behaviors (ILRs)*						
Time effect	0.04	0.96	0.02	0.28	0.76	0.02
Group effect	1.85	0.17	0.10	1.00	0.38	0.07
Interaction time x group	**8.11**	**< 0.01**	**0.32**	**8.91**	**< 0.01**	**0.40**
*Recovery during sleep (HRV)*						
Time effect	1.05	0.36	0.06	1.58	0.22	0.10
Group effect	4.49	0.02	0.20	1.19	0.32	0.08
Interaction time × group	0.55	0.58	0.03	0.29	0.75	0.02
**Repeated-measures ANOVA**						
*ILR*1:* Sleep/time awake*						
Time effect	0.71	0.79	0.002	0.52	0.48	0.02
Group effect	3.77	0.06	0.10	1.66	0.21	0.06
Interaction time × group	**10.00**	**< 0.01**	**0.22**	**10.87**	**< 0.01**	**0.28**
*ILR*2:* Physical activity/inactivity*						
Time effect	0.008	0.93	0.00	0.46	0.51	0.02
Group effect	1.88	0.18	0.05	0.27	0.61	0.01
Interaction time × group	0.03	0.87	0.001	0.14	0.71	0.005
*RMSSD*						
Time effect	1.84	0.18	0.05	1.37	0.25	0.05
Group effect	0.20	0.66	0.005	0.11	0.74	0.004
Interaction time × group	0.35	0.56	0.009	0.29	0.59	0.01
*SDNN*						
Time effect	0.16	0.70	0.004	0.02	0.89	0.001
Group effect	1.46	0.23	0.04	1.31	0.26	0.04
Interaction time × group	0.35	0.56	0.009	0.00	0.99	0.00

ηₚ² = partial eta squared. ILR1 = sleep relative to time awake, ILR2 = time being active relative to time being inactive during time awake. RMSSD and SDNN=heart rate variability (as a proxy for recovery during sleep). The adjusted model is adjusted for BMI, age, sex, and the presence of children living at home. Statistically significant effects (*p* < 0.01) are indicated in bold

## Discussion

This study aimed to examine the effects of a co-created workplace intervention among office workers with FWAs on the 24-hour composition of physical behaviors (i.e., time spent in sleep relative to time awake, ILR1, and time awake being physically active relative to being inactive, ILR2) as well as physiological recovery during sleep. The intervention combined individual- and workgroup-level strategies intended to promote recovery. Overall, we found the intervention to increase time spent in sleep relative to time awake (strong and statistically significant interaction between time and group for ILR1), while we did not find statistically significant intervention effects on physical activity relative to inactivity (ILR2) or on recovery during sleep (RMSSD and SDNN).

The descriptive results indicate a substantial increase in sleep duration among participants in the intervention group. On average, they increased their sleep time by 36 min from baseline to follow-up, whereas the control group showed a decrease of 23 min. The increase in sleep duration in the intervention group to 412 min, i.e. almost 7 h, is notable when compared to clinical evidence. For example, a meta-analysis of randomized controlled trials in patients with primary sleep disorders found moderate evidence of a 5–25-minute increase in sleep duration and a reduction of sleep latency by on average 7 min after applying melatonin compared to placebo [59]. Thus, our findings suggest that the intervention may have contributed to a clinically relevant, more favorable balance between sleep and time awake, bringing the intervention group closer to the recommended 7–8 h of sleep per night, which is likely to promote recovery and support overall health [60, 61].

The intervention did not, however, significantly increase HRV during sleep according to our statistical analyses. A significantly increased HRV during sleep would have reflected greater parasympathetic activity implying more efficient recovery [29]. Thus, although the intervention led to longer sleep time, this was not accompanied by detectable changes in physiological recovery according to HRV. This may be explained by the fact that the intervention was designed to target individual and collective work practices in flexible work, rather than directly targeting autonomic regulation. It is also possible that HRV is less sensitive to the present type of intervention, that physiological changes require more time, or that the small sample limited the ability to detect changes. We also noted a considerable variability between participants in HRV measurements, and it may be that our HRV data were influenced by individual differences in, e.g. day-to-day fluctuations, and/or external stressors [48], which can have masked a likely small but meaningful effect of the intervention.

Furthermore, the intervention did not change the ratio of time spent in physical activity relative to inactivity (ILR2). The descriptive data (Table 2), suggest that the increased time spent sleeping replaced both inactive and active behaviors during waking hours, while their relationship remained largely the same due to the properties of compositional data analysis. While physical activity may be influenced indirectly through improved detachment [9, 22], the intervention was primarily aimed at supporting psychological detachment from work, and not specifically at promoting physical activity or decrease sedentary behavior. The analysis of physical behaviors should therefore be considered exploratory, taking advantage of the available accelerometry data to examine whether the intervention was associated with changes in the overall 24-hour composition of physical behaviors. As shown in previous research, achieving measurable changes in physical activity or sedentary behavior in office settings may require targeted intervention activities focusing on increasing physical activity [62, 63]. For example, interventions may include instructions on how to incorporate more physical activity during a workday (e.g., by active transport, walk and talk meetings, and breaks in sitting time) [63] or how to utilize flexibility policies to incorporate physical activity into the day [62].

The current results differ from previous findings on psychological indicators of recovery, where the same intervention facilitated psychological detachment and reduced the need for recovery among employees in the intervention group with high initial need for recovery [39]. Physical activity and sleep duration reflects behavioral aspects of recovery [9]. HRV during sleep, in turn, reflects physiological recovery processes [29]. These outcomes combined suggest that the intervention did support psychological and behavioral indicators of recovery, but that this was not sufficient to influence physiological recovery.

The intervention was developed through a co-creation approach, in which intervention activities were selected based on what the target group and employer considered feasible, relevant, and potentially effective in their work context [41], the latter based on the theoretical assumption that changes in individual and collective work practices could reduce work-related demands, facilitate psychological detachment from work, and thereby support recovery [28]. Previous intervention studies aiming to promote physiological recovery have included activities that actively facilitate relaxation [50], for example, mindfulness techniques, slow breathing, and recovery training [38, 64, 65]. While such activities were not selected as intervention components in the co-creation process, they may be relevant to consider in future workplace interventions targeting physiological recovery among office workers with FWAs.

### Strengths and limitations

A main strength of this study is the co-created and participative approach, which enabled us to implement relevant and feasible intervention activities that were attractive to the employees and the organization [41]. Initiatives based on co-creation and participation are generally recommended in research for implementing relevant interventions and increasing the chances of achieving the desired effects [35, 66]. Also, the implementation of intervention activities at multiple organizational levels may increase the potential for sustainable changes, compared to individual focused strategies that largely rely on behavior change [66]. Another strength is the use of technical measurements of physical behaviors and recovery during sleep, which provide complementary information on behaviors and recovery that are not captured by self-reported indicators [67].

However, the study also has limitations. While co-creation and participation are generally important for identifying interventions that have a reasonable likelihood of being implemented, a resulting consequence is that the interests of researchers are sometimes compromised by the interests of the participating organization. This may limit the generalizability of the results to other settings or occupational groups. Also, a randomized selection process is generally recommended in intervention research [68], but this was not feasible in our study as the intervention had to be planned in keeping with restrictions set up by the organization. The intervention and control groups consisted of two separate departments within the same division of the organization. This may have allowed participants in the two groups to talk to each other about the intervention, even though the departments were geographically separated. This may, in turn, increase the risk of bias due to contamination between the groups [69]. Another limitation is the small sample size and the limited number of measurement days, which may reduce the statistical power in our analyses and increase the risk of missing meaningful effects [70]. On the same note, intervention effects may be more pronounced among individuals with a higher initial need for recovery. In a previous publication based on the same intervention study, we found positive effects on self-reported psychological indicators of recovery, including need for recovery and psychological detachment among participants with particular need for recovery [39]. In the present analysis based on fewer participants, the small sample size did not allow us to conduct sensitivity analyses to explore intervention effects in particular sub-groups [28, 71]. Finally, our method for assessing sleep periods, i.e., by self-reports in the diary, did not allow for a detailed examination of sleep sufficiency and specific sleep patterns, such as whether awake periods during the night or daytime napping occurred, both of which are associated with the extent and quality of sleep [72]. Therefore, the present findings regarding sleep time should be interpreted with some caution, as they reflect self-reported sleep periods rather than objectively measured sleep duration.

### Recommendations for future research

Future studies, ideally with larger sample sizes, should explore effects on physiological recovery in more detail, including biological markers like cortisol levels or blood pressure, which can indicate allostatic load and explain effects on physiological recovery [73]. Future studies should also consider longer measurement and follow-up periods [74] and include further indicators of sleep quality that are important for health outcomes [60, 75].

## Conclusion

We found that office workers with flexible work arrangements increased their time sleeping relative to time awake after receiving an intervention comprising an individual-level course in digital tools and work strategies and a workgroup-level workshop to develop common rules and routines for flexible work, compared to a control group working as usual. The intervention did not influence physical activity relative to inactivity during time awake, nor heart rate variability during sleep. The results suggest that co-created workplace interventions targeting multiple organizational levels can support meaningful improvements in sleep when designed according to employee needs and implemented effectively in the workplace.

## Data Availability

The datasets analyzed in the present study are available from the corresponding author upon reasonable request.

## Abbreviations

BMI: Body mass index
CoDA: Compositional data analysis
FWAs: Flexible work arrangements
HR: Heart rate
HRV: Heart rate variability
ICT: Information and communication technology
ILR: Isometric log ratio
RM-ANOVA: Repeated-measures univariate analysis of variance
RM-MANOVA: Repeated-measures multivariate analysis of variance
RMSSD: Root mean square of successive differences between normal-to-normal intervals
RQ: Research question
SD: Standard deviation
SDNN: Standard deviation of inter-beat intervals of normal sinus beats
